# Sphingosine-1-Phosphate Promotes the Persistence of Activated CD4 T Cells in Inflamed Sites

**DOI:** 10.3389/fimmu.2017.01627

**Published:** 2017-11-24

**Authors:** Shafqat Ahrar Jaigirdar, Robert A. Benson, Aziza Elmesmari, Mariola Stefania Kurowska-Stolarska, Iain B. McInnes, Paul Garside, Megan K. L. MacLeod

**Affiliations:** ^1^Centre for Immunobiology, Institute of Infection, Immunity and Inflammation, University of Glasgow, Glasgow, United Kingdom

**Keywords:** inflammation, CD4 T cell, sphingosine-1-phosphate, migration, intravital imaging, rheumatoid arthritis

## Abstract

Inflammation can be protective or pathogenic depending on context and timeframe. Acute inflammation, including the accumulation of CD4 T cells, accompanies protective immune responses to pathogens, but the presence of activated CD4 T cells at sites of inflammation is associated with chronic inflammatory disease. While significant progress has been made in understanding the migration of CD4 T cells into inflamed sites, the signals that lead to their persistence are poorly characterized. Using a murine ear model of acute inflammation and intravital two-photon imaging, we have dissected the signals that mediate CD4 T cell persistence. We report the unexpected finding that the bioactive lipid, sphingosine-1-phosphate (S1P), is both necessary and sufficient for the persistence of activated CD4 T cells at peripheral tissues in acute inflammation. S1P mediated the enhanced motility of CD4 T cells at inflamed tissues but did not affect their migration to the downstream draining lymph node. We found that sphingosine kinase-1, which regulates S1P production is increased at inflamed sites in mice and in patients with the chronic inflammatory disease, rheumatoid arthritis. Together, these data suggest that S1P, or its regulators, may be key targets to promote or disrupt accumulation of CD4 T cells at inflamed tissues.

## Introduction

The traffic of T cells through lymphoid organs, blood, and inflamed tissues is a highly regulated process. This ensures that each unique T cell surveys antigen presented by dendritic cells (DCs) in all lymphoid organs and that activated T cells migrate to sites of infection to mediate pathogen control. While the migration of T cells to sites of infection is protective, their sustained recruitment or retention can lead to chronic inflammation.

Recognition of cognate antigen by T cells in lymphoid organs leads to their arrest. Once fully activated, T cells exit lymph nodes (LNs), a process regulated in part by the signaling sphingolipid, sphingosine-1-phosphate (S1P) (1). Activated T cells then migrate to inflamed tissues under the control of various signals including cell adhesion molecules, tissue addressins, chemokines, and S1P (2, 3). While these processes have been extensively studied, the molecular signals that mediate the persistence of activated CD4 T cells at sites of inflammation are poorly characterized.

Many activated CD4 T cells in inflamed sites undergo apoptosis. A minority of cells will turn into tissue-resident memory (Trm) cells, and in some cases, inflamed sites will become chronically inflamed. The sequence of molecular events leading to these distinct decisions is largely unknown. Expression of the chemokine receptor, CCR7, by T cells in peripheral tissues can prompt their exit into lymphatics and migration to LNs (4). Furthermore, growing evidence suggests that differentiation into resting Trm cells involves downregulation of one of the four receptors for S1P (S1PR1) and upregulation of CD69 (5, 6). These two molecules antagonize each other: S1PR1 promotes exit into S1P rich lymphatics while CD69 enhances retention by blocking S1PR1 surface expression.

By contrast, in chronic inflammation, continued activation, recruitment, and persistence of immune cells are accompanied by changes to the inflamed tissue itself. This includes increased vascularization and alterations to local stromal cell populations that can further enhance inflammation and promote activation of infiltrating immune cells (7–9).

Here, we investigate the molecular signals that regulate the residency time of activated CD4 T cells at inflamed sites. Using flow cytometry to quantify CD4 T cells and multiphoton laser scanning microscopy to analyze their movement behaviors *in vivo*, our data demonstrate an unexpected pivotal role for S1P in promoting the persistence of activated CD4 T cells in tissues.

Furthermore, we show that sphingosine kinase-1 (SPHK1), one of the enzymes responsible for the conversion of sphingosine into S1P, is increased at sites of acute inflammation in mice and in the inflamed synovium of rheumatoid arthritis (RA) patients. Our data suggest that increased production of S1P modulates CD4 T cell behavior affecting the persistence of CD4 T cells in sites of acute inflammation. Understanding these processes will have important implications for therapeutic interventions that seek either to increase T cell persistence at tissue sites following vaccination or reduce the accumulation of T cells in chronic inflammation.

## Materials and Methods

### Animals

6-week-old male C57BL/6 mice (Envigo) were used following 1 week of acclimatization. Transgenic mice: CD45.1+ OT-II T cell receptor transgenic mice (10); OT-II mice crossed to hCD2-DSRed (original gifted by gifted by D. Kioussis and A. Patel, National Institute for Medical Research, London); CD11c-YFP (11); and LysMGFP (12) (originally gifted by Sussan Noursargh, William Harvey Research Institute) were bred in house. All mice were housed at the University of Glasgow and maintained under standard animal husbandry conditions. Experiments were covered by a Project License granted by the UK Home Office under the Animals (Scientific Procedures) Act of 1986 and approved by the University of Glasgow Ethical Review Committee.

### CD4 T Cell Polarization

CD4 T cells were isolated by negative selection (STEMCELL Technologies) from spleens and LNs of OT-II mice and cultured with mitomycin c (Sigma-Aldrich) treated splenocytes in T helper 1 (Th1) conditions [1 µg/ml Ova Peptide_323–339_ (Sigma-Aldrich), 10 µg/ml anti-IL4 (BioXcell), and 20 ng/ml IL-12 (R&D systems)] for 3 days in complete RPMI 1640 (Gibco) (10% FCS, 100 µg/ml penicillin–streptomycin and 2 mM l-glutamine) at 37°C/5% CO_2_.

### CD4 T Cell Treatments and Fluorescent Labeling

Polarized CD4 T cells were treated with pertussis toxin (PTX) (100 ng/ml) (Sigma-Aldrich), FTY720 (Sigma-Aldrich) (0.5 µg/ml), SEW2871 (R&D Systems) (5 µg/ml), W146 (R&D Systems) (5 µg/ml) or vehicle for 60 min (PTX) or 90 min in complete RPMI at 37°C/5% CO_2_. For proliferation studies, CD4 T cells were labeled with cell tracker blue (Thermo-Fischer Scientific) at 5 µM for 15 min at 37°C. After all treatments, cells were washed thoroughly before transfer.

### Intradermal Lipopolysaccharide (LPS) Administration and CD4 T Cell Adoptive Transfer

10 µg of LPS from *E. coli* (Sigma-Aldrich, Strain 0111:B4) was injected intradermally in 10 µl into the ear pinna. 1–3 × 10^6^ *in vitro* polarized CD4 T cells were transferred intradermally into the same injection site. For intravital microscopy studies, 2 × 10^6^ CD4 T cells in 2 µl were injected intradermally at shallow depths to allow visualization. Ear pinnae thickness was measured using digital calipers (Kroeplin GmbH, model C1X018).

### Tissue Preparation

Mouse ears and lymphoid organs were harvested and single cell suspensions prepared. Ears were digestion in 2 mg/ml Collagenase IV (Sigma-Aldrich), 2 mg/ml hyaluronidase (Sigma-Aldrich), and 100 U/ml DNase I (Invitrogen) at 37°C for 40 min at 180 RPM in a rotating incubator. Following digestion, a single cell suspension was prepared with a gentlemacs dissociator (Miltenyi Biotec) in a gentlemacs C tube (Miltenyi Biotec). Viable cells were counted on a hemocytometer with dead cells excluded by trypan blue. LNs and spleens were disrupted into a single cell suspension between two pieces of 40 µm nitex.

### Flow Cytometry

Single cell suspensions were incubated with a fixable viability dye (eBioscience) for 20 min at 4°C. Samples were blocked with FC block (24G2 grown in house and mouse serum) for 20 min followed by antibody staining for 20 min. Antibodies used: CD45.1 (A20, eBioscience), CD4 (RM4-5, eBioscience), Va2 (B20.1, BD), MHC II (M5/114.15.2, eBioscience), CD64 (X54-5/7.1, BioLegend), CD8a (53-6.7, eBioscience), CD103 (M290, BD Horizon), Ly6G (1A8 BD), CD69 (H1.2F3, BD), S1PR1 (713412, R&D Systems), interferon-γ (IFN-γ) (XMG1.2, BioLegend), and CD44 (IM7, eBioscience). Samples were washed twice with FACS buffer and acquired on a Miltenyi Macsquant analyzer. Samples were analyzed using FlowJo (Treestar) version 9.7.5.

### Multiphoton Laser Scanning Microscopy

A Zeiss LSM 7MP system equipped with 20×/1.0 NA water-immersion objective lens (Zeiss UK, Cambridge, UK) and a tunable titanium: sapphire solid-state two-photon excitation source (Chameleon Ultra II; Coherent Laser Group, Glasgow, UK) and optical parametric oscillator (Coherent Laser Group) were used. Animals were anesthetized with 10 mg/kg ketaset mix administered intraperitoneally. The ear was immobilized on a stand using veterinary grade glue and the animal’s core temperature maintained using a heat mat. Videos were acquired in 15–30 min intervals at an X–Y pixel resolution of 512 × 512 with 1.5 µm increments in Z stack. Videos were analyzed with Volocity version 6 after correction for tissue drift using second harmonic as the anchor. Individual cells were defined as objects and tracked in 3D. Cells were identified using intensity thresholding and object volume. Track plots are included to demonstrate the actual migration of cells relative to their point of origin. Displacement rate was defined as the displacement/(time of last point on track − time of the first time point on the track) giving a normalized view of how far individual cells have traveled. Meandering index (also known as confinement ratio or chemotactic index) is a ratio defining track straightness. It is defined as the ratio of displacement of the cell to the total length of the track, with 0 being a highly confined cell that returns to its starting position, and 1 being a cell traveling in a completely straight line.

### Patient Samples

Synovial tissue specimens were obtained from RA and osteoarthritis (OA) patients during arthroscopic biopsy or total joint replacement surgeries at Glasgow Royal Infirmary (Glasgow, UK). All RA and OA patients fulfilled the diagnostic criteria for RA and OA classification respectively, and written consent form was obtained from all subjects. All procedures received Ethics Approval (West of Scotland Research Ethical Committee Approval: 11/S0704/7).

### Mouse and Human Tissue Staining and Preparation

Mouse ears or human synovial tissues were preserved in 10% formalin, paraffin embedded and sectioned (10 or 5 µm, respectively). Tissues were hydrated through xylene and alcohols and the peroxidase activity blocked. Antigens were retrieved by boiling in antigen retrieval buffer (Abcam). Samples were blocked with 2.5% horse serum and an avidin/biotin kit (Vector Labs). Tissues were stained with rabbit polyclonal anti-SPHK1 (ab16491, Abcam) at 2.5 µg/ml overnight in a dehumidified chamber at 4°C. Biotinylated anti-rabbit (1:200) (Vector Labs) in 2.5% horse and mouse/human serum, respectively, was added and incubated for 30 min at room temperature and streptavidin-PE (mouse) or avidin/biotin complex (human) (Vector Labs) added to the sections for 30 min. Human sections were washed and dried before 3,3′-diaminobenzidine (Vector Labs) was added. The sections were developed before washing, dehydration and mounting in DPX. Mouse sections were mounted with DAPI+ prolong gold. Tissues were imaged using an Olympus BX 41 microscope attached with a DP 25 camera with axiovision software at 10× or 20× (human) or a Cell Observer SD (Zeiss) (mouse). The staining in synovial lining, sub-lining and vascular endothelial cell layers were graded on a scale of 0–4 (0 = no cells, 1 = <25%, 2 = <25–50%, 3 = < 50–75%, 4 = 75%) by two blinded observers on two independent occasions. Ear sections from individual animals were imaged using a tile scan and analyzed using ImageJ software (NIH). Three equally sized areas were randomly selected per section, and the number of total and SPHK1+ cells manually counted.

### Statistical Analysis

All data shown were analyzed using GraphPad Prism version 6. Error bars are SEM. Mann–Whitney test (two groups) or one-way ANOVA statistical analysis tests (more than two groups) were carried out with Tukey’s multiple comparison tests (ANOVA only) to compare the differences between groups. *Denotes *p* values of <0.05, **p < 0.01, ***p < 0.001, ****p < 0.0001.

## Results

### More Activated CD4 T Cells Are Retained at Inflamed than Resting Tissue Sites

The pool of T cells found at inflamed sites includes newly recruited cells and those that are retained, making it difficult to separate the signals that cause recruitment from those responsible for persistence. To address this, we transferred a congenic (CD45.1+) population of CD4 T cells directly into the ear pinnae of mice. By analyzing and manipulating these cells, we have developed a system to determine the signals that regulate CD4 T cell persistence without the confounding factor of ongoing recruitment.

**Figure 1:**
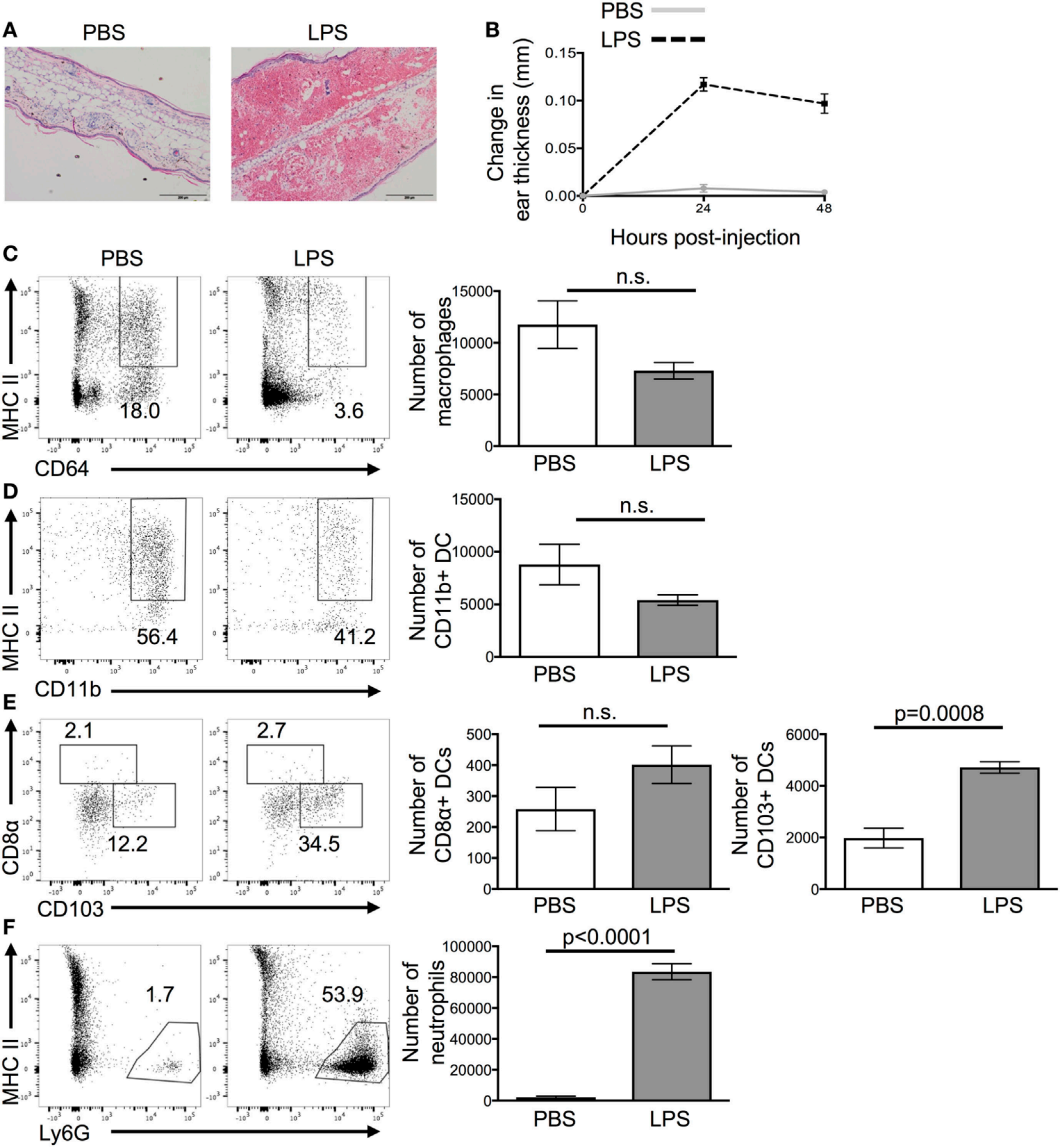
Lipopolysaccharide (LPS) injection into the ear causes extensive cellular infiltrate. 24 h after the injection of PBS or LPS into ear pinnae, the response was examined by microscopy **(A)**, digital calipers **(B)**, or flow cytometry **(C–F)**. Representative images of H&E stained sections **(A)** or homogenized ears **(C,D)** are shown. In panel **(A)**, the scale bar shows 200 μm. In panel **(B)**, an average of three individual measurements was taken for each ear pinnae. In panels **(C,E)**, cells are gated on live cells, in panel **(D)** on live CD45+ CD11c+ cells and in panel **(F)** on live CD45+ CD11c+ MHCII+ cells. Data are representative of two independent experiments with three to five animals per group. Error bars show SEM.

Ear pinnae were inflamed by injecting LPS intradermally into the tissue as it causes reproducible acute inflammation (Figure 1A). Peak inflammation in the ear was evident 24 h after LPS injection; we, therefore, focused on this time point (Figure 1B). Analysis of the cellular infiltrate by flow cytometry showed a large influx of neutrophils and CD103+ DCs but no change in the numbers of CD11b+ DCs, CD8α+ DCs, or macrophages (Figures 1C–F).

Activated rather than naïve CD4 T cells are found in inflamed peripheral tissues (13). We, therefore, transferred CD4 OT-II T cells differentiated into Th1 cells *in vitro* (14) into either resting (PBS injection) or LPS-inflamed ear pinnae 24 h after the initial LPS injection. Typically, 80%+ of the activated OT-II T cells expressed IFN-γ, the signature cytokine for Th1 cells (Figure S1 in Supplementary Material).

**Figure 2:**
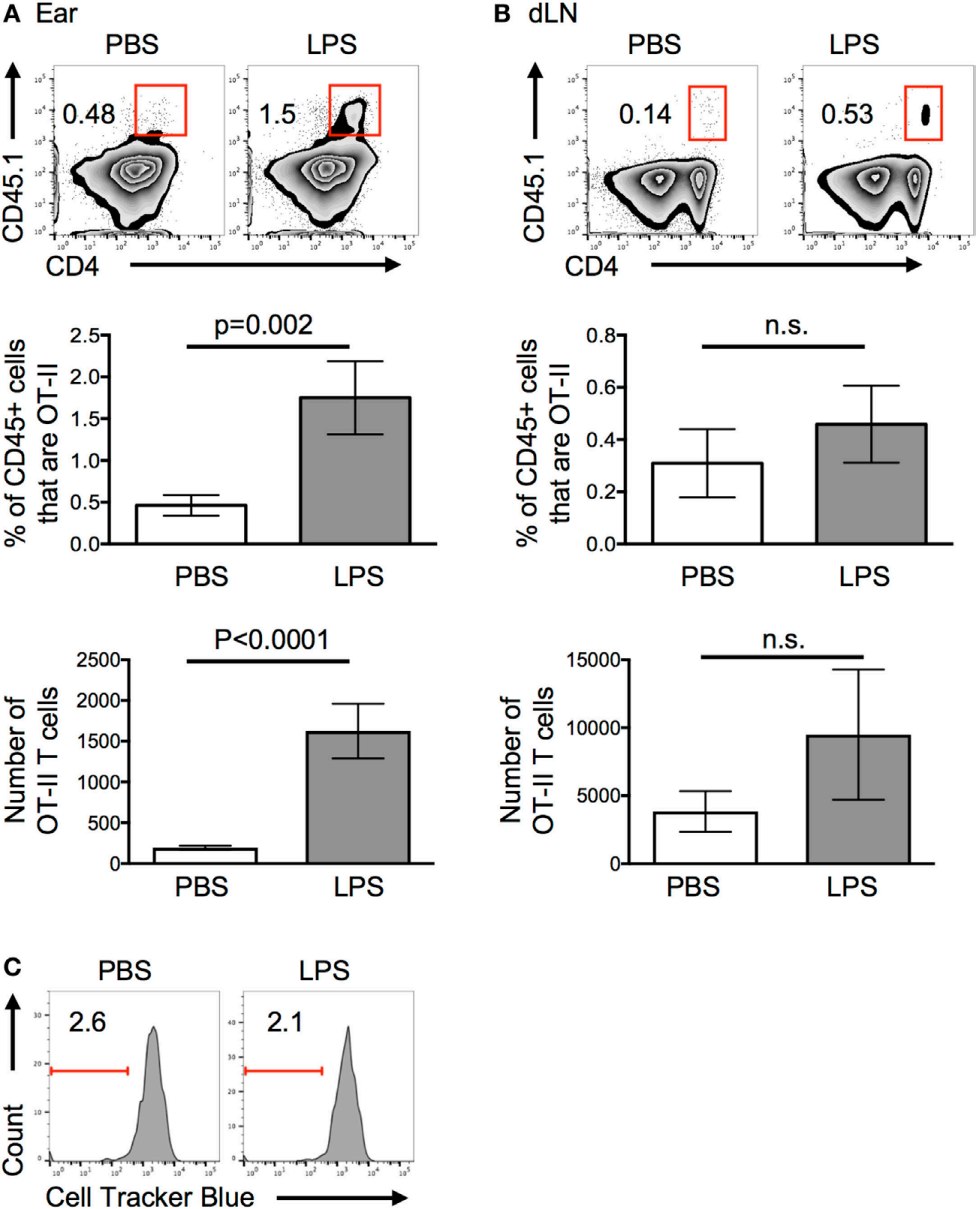
Lipopolysaccharide (LPS)-induced inflammation increases the persistence of activated T cells in inflamed sites. Activated OT-II T cells were transferred into the ear pinnae of mice injected with PBS or LPS 24 h previously. After a further 24 h, the percentages and numbers of OT-II T cells in the ears **(A)** or downstream draining lymph nodes **(B)** were examined by flow cytometry and their proliferation **(C)** assessed. In panel **(C)**, OT-II T cells were labeled with cell tracker blue before transfer. In panels **(A,B)**, cells are gated on live CD45+ cells and in panel **(C)** on live CD45+ cells that are CD4+ CD45.1+ (OT-II). Data are representative or combined from three independent experiments with ≥3 animals per group. Error bars are SEM.

More OT-II T cells were recovered from inflamed compared with control ears 24 h after cell transfer (Figure 2A). By contrast, similar numbers of OT-II T cells were found in the draining LN (Figure 2B). Very few, if any, OT-II T cells were found in the contralateral cervical LNs and none were found in the spleen (Figure S2 in Supplementary Material). The striking difference in the recovery of cells in inflamed versus resting tissues 24 h after cell transfer, convinced us to focus on the factors that contributed to this early increase in CD4 T cell persistence in inflamed tissues.

### Proliferation Does Not Contribute to the Increase in Number of CD4 T Cells at the Inflamed Site

Increased persistence of OT-II T cells at the inflamed site could be due to decreased migration to the draining LN, proliferation at the site, or increased survival. As we found no significant decrease in the numbers of CD4 T cells in the LN downstream of the LPS-injection site (Figure 2B), we can conclude that OT-II T cells did not migrate from the inflamed tissue at a slower rate. Elevated numbers in the ear may have been due to either proliferation to increase their number and/or increased survival. Transfer of OT-II T cells labeled with a fluorescent cell tracker dye demonstrated that OT-II T cells did not proliferate in either resting or inflamed ears (Figure 2C). Assays to measure signs of apoptosis within the transferred cells proved inconclusive, most likely because of the extended processing required to isolate cells from the ears.

### Inflammation Alters the Behavior of Activated CD4 T Cells *In Vivo*

In secondary lymphoid organs, interactions between CD4 T cells and other immune cells, especially antigen presenting DCs, can alter their migration patterns (15). Although specific antigen is not present in our *in vivo* model, the influx of class II positive DCs into inflamed ears, could lead to a reduction in cell motility that consequently affected their persistence. To address these questions, we imaged PBS or LPS-injected ear pinnae 4–5 h following transfer of fluorescently labeled activated OT-II T cells. This early time point enables us to identify cell behaviors that may indicate why we find increased numbers of OT-II T cells in LPS-inflamed ear pinnae 24 h after cell transfer.

**Figure 3:**
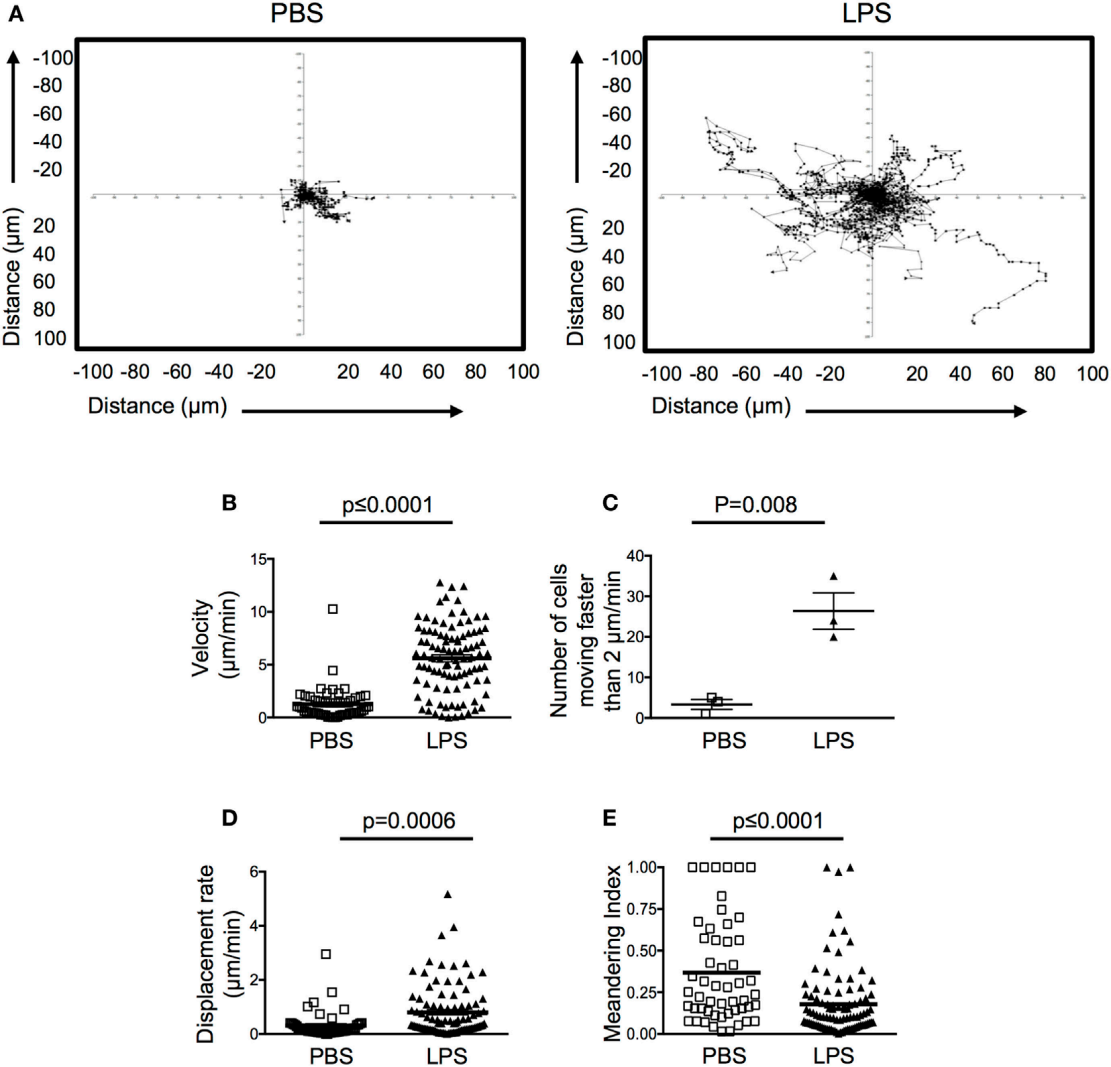
Activated CD4 T cells have increased velocity and displacement rate in lipopolysaccharide (LPS)-inflamed sites. Activated OT-II T cells were transferred into the ear pinnae of mice injected with PBS or LPS 24 h previously. After a further 24 h, mice were anesthetized, and OT-II T cells imaged by intravital multiphoton microscopy. Data show tracks of individual cells from the ear of one mouse per treatment **(A)** or combined data to show velocity **(B)**, number of cells per mouse moving above 2 μm/min **(C)**, displacement rate **(D)** or meandering index **(E)**. In panels **(B,D,E)**, each symbol represents a cell, and the horizontal line indicates the mean of the group, in panel **(C)** each symbol represents a mouse, and the horizontal line shows the mean of the group; error bars are SEM. Three animals were analyzed per treatment.

Motile behavior of the transferred OT-II T cells in the resting ear pinnae was restricted, demonstrating low speeds and displacement rate (Figures 3A–C; Videos S1 and S2 in Supplementary Material). Surprisingly, in inflamed ears, OT-II T cells demonstrated higher motility and more cells moved faster than 2 μm/min, below which cells are considered to have arrested their movement (16). OT-II T cells in inflamed tissues also had an increased displacement rate compared with the OT-II T cells in PBS-injected ears (Figures 3A–D). The displacement rate of the OT-II T cells in LPS-injected ears was similar to that of T cells in LNs or peripheral tissues and suggests that the cells are not confined in the inflamed tissue (17, 18). The OT-II T cells in inflamed ears did not display directional bias as indicated by their low meandering indexes (Figure 3E). Such highly motile behavior is suggestive of a lack of stable cellular interactions. Consistent with this, the OT-II T cells were not seen to form stable contacts with infiltrating DCs (Videos S1 and S2 in Supplementary Material).

### S1P Promotes Persistence of Activated CD4 T Cells at Inflamed Tissue Sites

**Figure 4:**
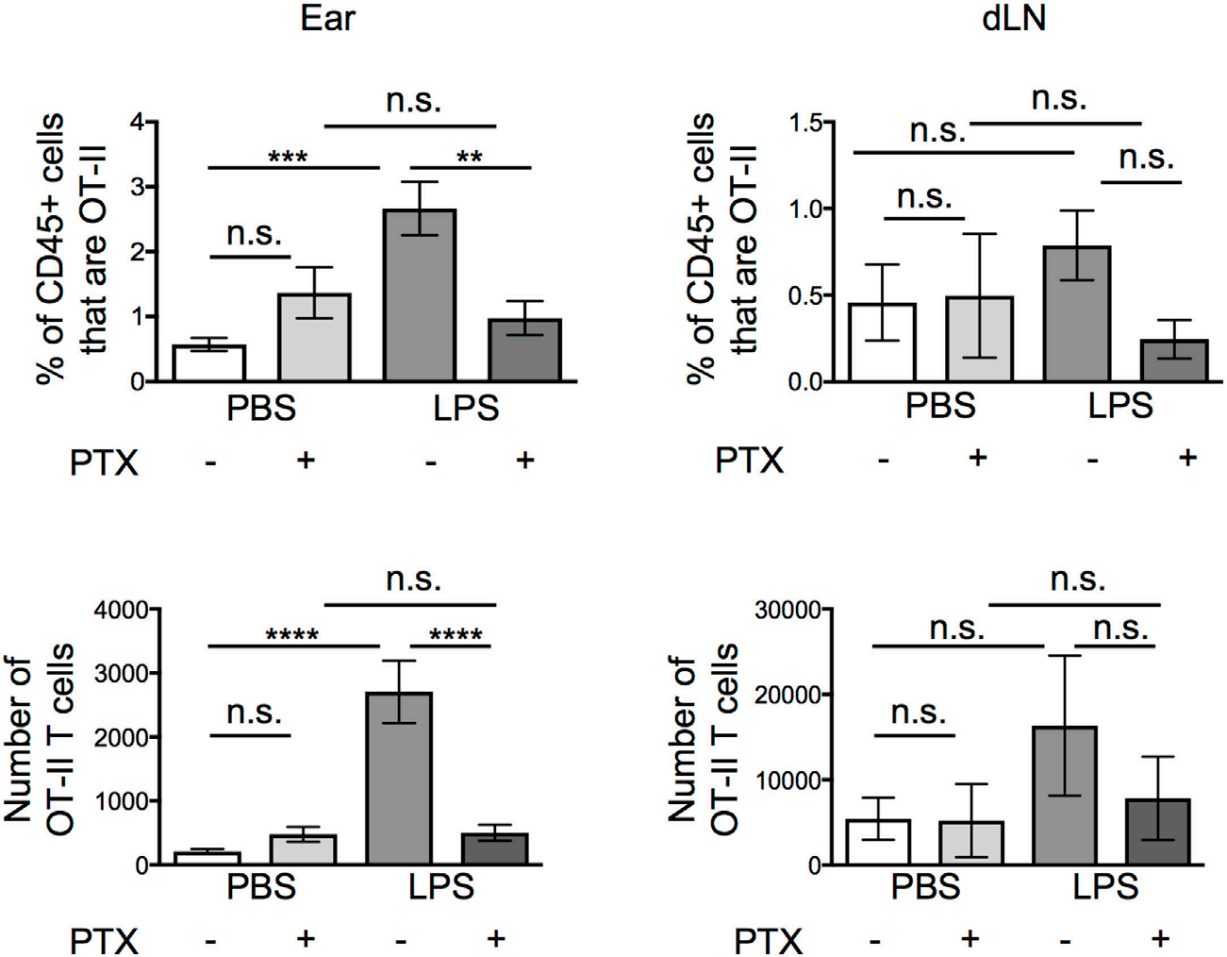
Signals through G protein-coupled receptors cause increased persistence of activated CD4 T cells at lipopolysaccharide (LPS)-inflamed sites. Activated OT-II T cells were treated with vehicle or pertussis toxin (PTX) before transfer into the ear pinnae of mice injected with PBS or LPS, 24 h earlier. After a further 24 h, the percentages and numbers of transferred OT-II T cells in the homogenized ear or draining lymph node were examined by flow cytometry. Data are combined from two to three independent experiments with ≥3 animals per group. In this figure, *p* < 0.01 is indicated by **; *p* < 0.001 is indicated by ***; and *p* < 0.0001 by ****; n.s., non-significant.

The lack of cellular interactions in the inflamed ears suggested that soluble mediator(s) may be more likely to be responsible for the increased persistence of the OT-II T cells. Many soluble mediators that regulate cell migration signal *via* G-protein coupled receptors. Signaling through these receptors can be blocked by treatment with Pertussis Toxin (PTX) (19–21). Before transfer, we treated activated OT-II T cells with vehicle or PTX and examined the percentages and numbers of OT-II T cells in the ear and in the downstream draining LN 24 h after transfer. While PTX did not alter the numbers of OT-II T cells that remained in resting ear pinnae, it greatly reduced the number of OT-II T cells in inflamed ears, without significantly increasing the number of OT-II T cells found in the draining LN (Figure 4).

**Figure 5:**
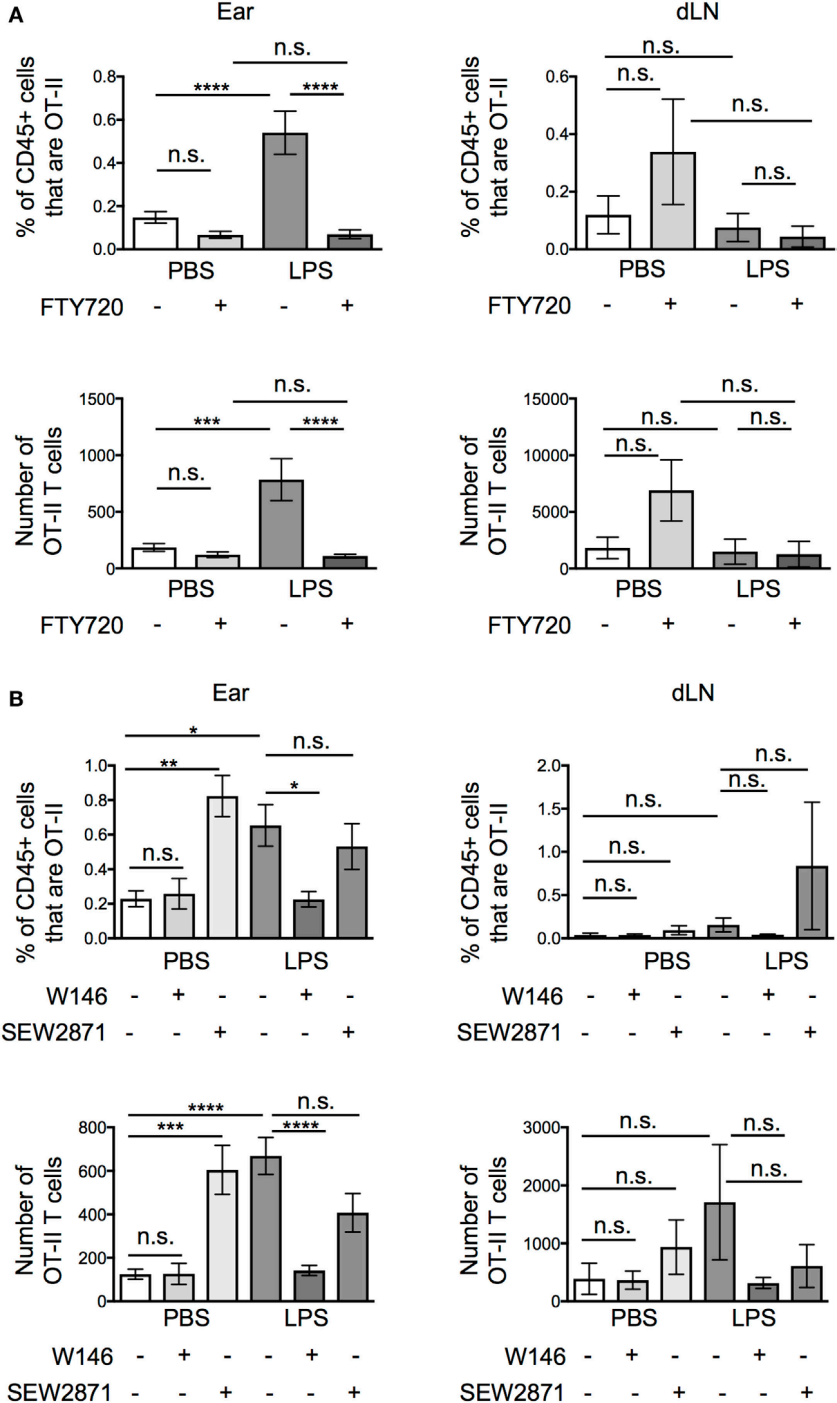
Signals through S1PR1 cause increased persistence of activated CD4 T cells at lipopolysaccharide (LPS)-inflamed sites. Activated OT-II T cells were treated with vehicle or FTY720 **(A)**, or S1P1 receptor specific antagonist (W146) or agonist (SEW2871) **(B)** before transfer into the ear pinnae of mice injected with PBS or LPS 24 h earlier. After a further 24 h, the percentages and numbers of transferred OT-II T cells in the homogenized ear or draining lymph node were examined by flow cytometry. Data are combined from two to three independent experiments with ≥3 animals per group. In this figure, *p* < 0.05 is indicated by *; *p* < 0.01 is indicated by **; *p* < 0.001 is indicated by ***; and *p* < 0.0001 by ****; n.s., non-significant.

Pertussis toxin blocks the activity of chemokine receptors and receptors for the sphingolipid, S1P, all of which are associated with cell migration (1, 19–21). To differentiate between roles for chemokines and S1P, we treated the activated OT-II T cells with FTY720, a functional antagonist of S1P receptors (1, 22), before transfer. Unexpectedly, FTY720 treatment greatly reduced the numbers of OT-II T cells at the inflamed tissue site, suggesting that S1P, rather than chemokines were responsible for the increased recovery of OT-II T cells from inflamed as compared with resting ear pinnae (Figure 5A). FTY720 treatment did not increase the numbers of T cells recovered from the downstream draining LN demonstrating that it did not increase T cell migration out of inflamed ears.

FTY720 causes internalization of S1P receptors, preventing cells from sensing S1P gradients *in vivo*. It is possible that *in vitro* FTY720 treatment caused some S1P receptor signaling. To confirm whether the effect we observed was due to agonist or antagonist effects of FTY720, we treated activated OT-II T cells with a specific S1PR1 antagonist, W146, or agonist, SEW2871. These agents act only on the S1PR1, the S1P receptor associated with the regulation of T cell exit from lymphoid organs (23, 24). Activated OT-II T cells pretreated with W146 phenocopied those treated with FTY720 with fewer OT-II T cells recovered from inflamed ears compared with ears injected with vehicle treated OT-II T cells (Figure 5B). By contrast, pretreatment of activated OT-II T cells with the S1P1R agonist, SEW2871, did not alter the numbers of OT-II T cells recovered from inflamed sites. SEW2871 pretreatment did, however, increase the recovery of OT-II T cells from resting ears to levels similar as from inflamed ears. These data demonstrate that signals through the S1P1R are sufficient to promote CD4 T cell persistence 24 h posttransfer (Figure 5B). Notably, neither treatment altered the numbers of OT-II T cells recovered from the draining LNs (Figure 5B).

### S1P1R on Activated OT-II T Cells Mediates Cell Movement *In Vivo*

**Figure 6:**
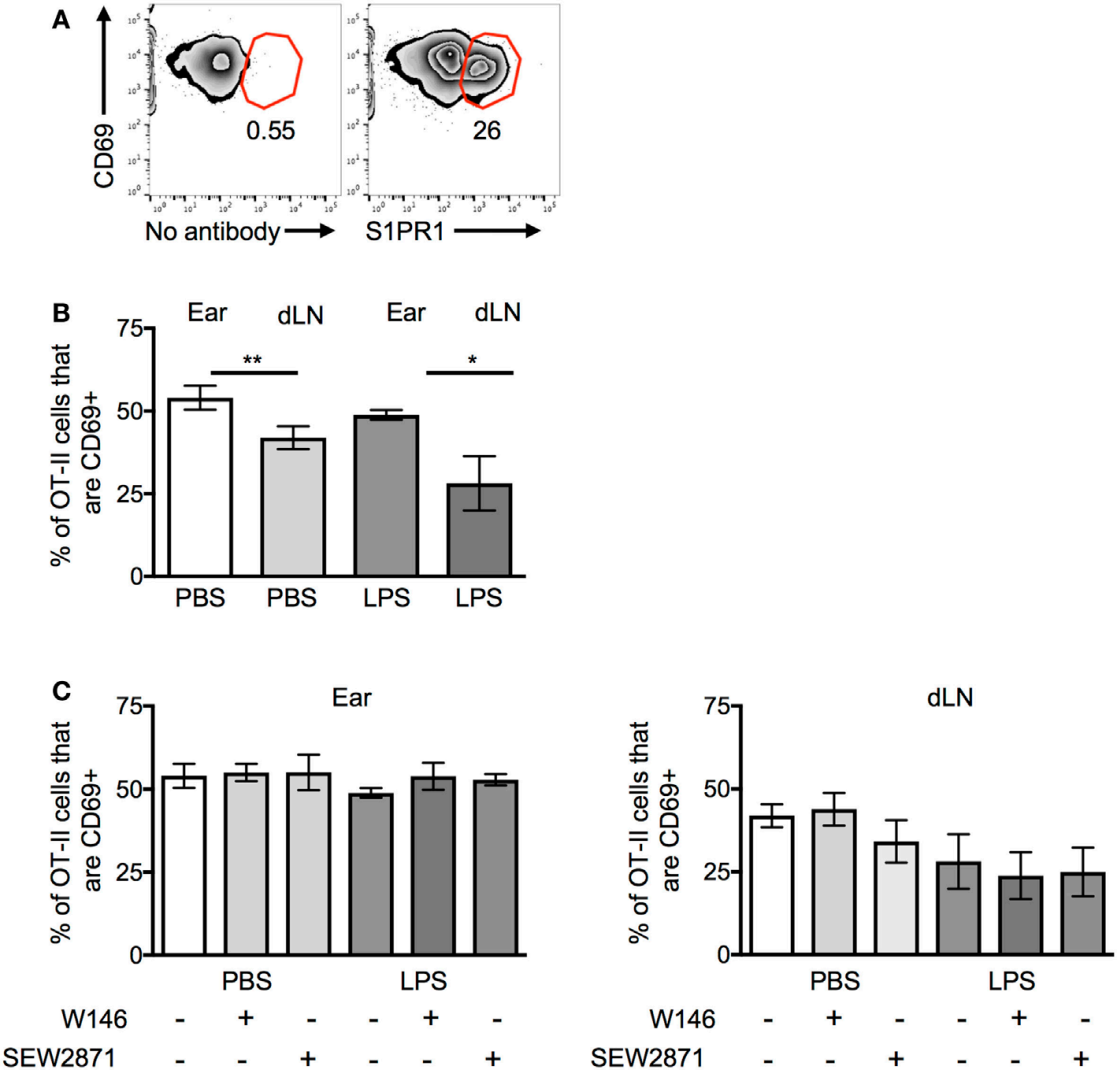
CD69 and S1PR1 expression on CD4 T cells before and following cell transfer. Activated OT-II T cells were analyzed before cell transfer **(A)** for expression of CD69 and S1PR1 or for CD69 expression *ex vivo* 24 h after transfer into resting (PBS) or inflamed [lipopolysaccharide (LPS)] ear pinnae **(B,C)**. Before transfer, the OT-II T cells were treated with an S1PR1 antagonist or agonist as in Figure 5B. In panel **(A)**, cells are gated on live CD4+ CD45+ cells; representative of three independent samples. In panels **(B,C)**, data are combined from two to three independent experiments with ≥3 animals per group. In this figure, *p* < 0.05 is indicated by * and *p* < 0.001 by **.

We were surprised that the OT-II T cells were responsive to S1P signals as S1PR1 is reported to be downregulated on recently activated T cells (22). Activated OT-II T cells before transfer expressed high levels of CD69, indicative of their recent exposure to antigen *in vitro*. However, a sizable proportion of the cells were also positive for S1PR1; these cells expressed slightly reduced levels of CD69 (Figure 6A).

OT-II T cells isolated from the ear pinnae expressed small but significantly higher levels of CD69 than those in the LNs, regardless of whether the ear had been inflamed or not (Figure 6B). However, neither treatment with S1PR1 antagonists nor the agonist affected CD69 expression (Figure 6C). These data suggest that the increased persistence of CD4 T cells at inflamed sites was not mediated *via* CD69 and that modulation of S1PR1 signaling did not affect the expression of CD69.

**Figure 7:**
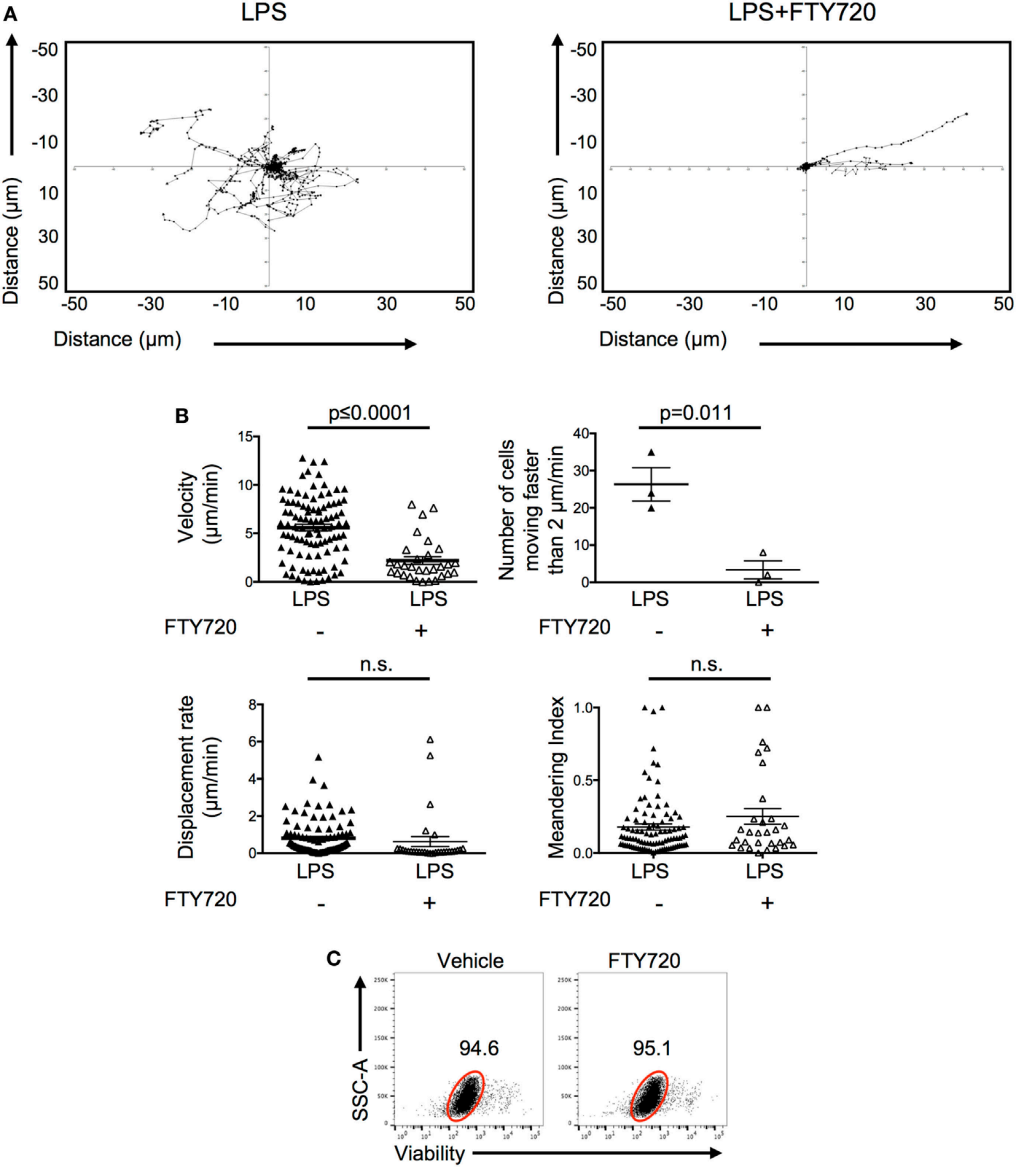
Signals through S1P1 receptor cause increased CD4 T cell movement in inflamed tissues. Activated OT-II T cells were treated with vehicle or FTY720 before transfer into the ear pinnae of mice injected with PBS or lipopolysaccharide (LPS) 24 h earlier. After a further 24 h, mice were anesthetized and OT-II T cells imaged by multiphoton microscopy. Data show tracks of individual cells from the ear of one mouse per treatment **(A)** or velocity, number of cells per mouse moving above 2 μm/min, displacement rate and meandering index. For velocity, displacement rate, and meandering index, each symbol represents a cell, and the horizontal line indicates the mean of the group. For numbers of cells moving above 2 μm/min, each symbol represents a mouse, and the horizontal line shows the mean of the group; error bars are SEM. Data are combined from three animals per group **(B)**. In panel **(C)**, activated OT-II T cells treated with vehicle or FTY720 were rested overnight *in vitro* and viability determine by exclusion of viability dye. Cells are gated on activated cells, and data are representative of three experiments.

To investigate how S1P promoted the persistence of activated OT-II T cells, we imaged the behavior of the FTY720-treated OT-II T cells in inflamed ears. Activated CD4 T cells pretreated with FTY720 and injected into LPS-inflamed ear pinnae had reduced cell velocity with fewer cells moving above 2 μm/min (Video S3 in Supplementary Material; Figures 7A,B) compared with control activated T cells. Their behavior was, therefore, similar to that seen in non-inflamed ears (Figure 3). FTY720-treated cells had similar displacement rates and meandering indices compared with control T cells transferred into LPS-inflamed ears suggesting either that FTY720 treatment did not fully inhibit S1P signal or that additional molecules, such as chemokines and adhesion molecules, are involved in regulating T cell motility within inflamed tissues (25, 26). Importantly, we did not observe any direct toxicity of FTY720 as the vast majority of FTY720-treated OT-II T cells cultured *in vitro* were viable 24 h later (Figure 7C). Together, these data indicate that S1P may act to increase CD4 T cell persistence by promoting CD4 T cell survival at the inflamed site.

### Inflamed Mouse and Human Tissues Have Increased Levels of the S1P Generating Enzyme, SPHK1

**Figure 8:**
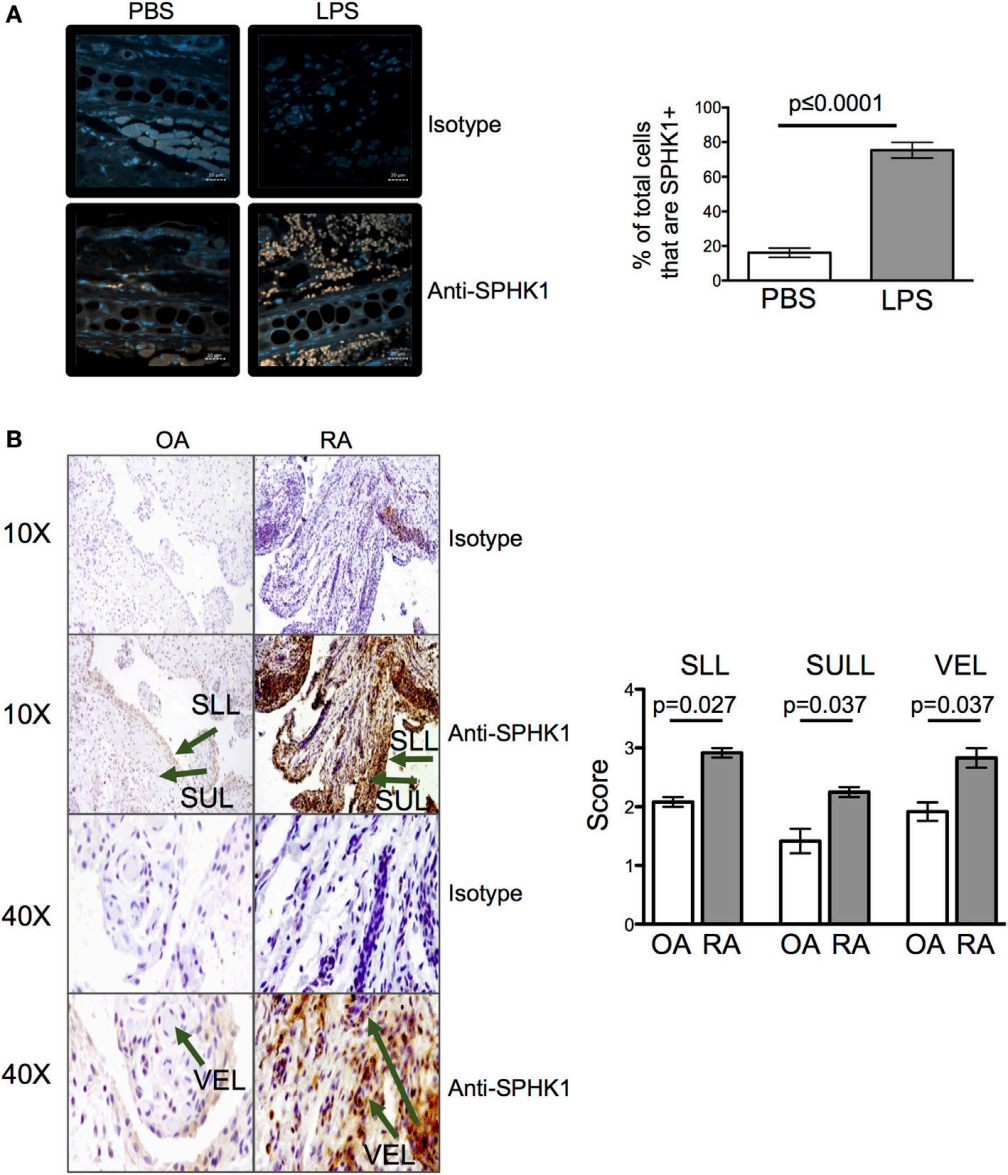
Inflammation leads to an increase in sphingosine kinase-1 (SPHK1). Levels of SPHK1 in the ear pinnae of mice injected with PBS or lipopolysaccharide (LPS) 24 h **(A)** previously or in sections prepared from osteoarthritis (OA) or rheumatoid arthritis (RA) patients **(B)** were examined. Sections were immunostained with anti-SHPK1 (brown) and DAPI (blue), scale bar in panel **(A)** is 20 µm **(A)**. Data are representative or combined from five animals per group or four OA and four RA patients. In panel **(B)**, SLL, synovial lining; SUL, synovial sub-lining; and VEL, vascular endothelial lining, and staining graded on a scale of 0–4 (0 = no cells, 1 = <25%, 2 = <25–50%, 3 = <50–75%, 4 = 75%) by two blinded observers on two independent occasions.

The levels of extracellular S1P available to cells in tissues are difficult to measure with any accuracy *ex vivo* (1). To determine whether inflammation may alter the amount of S1P at tissue sites, we quantified the levels of SPHK1, an enzyme that drives the conversion of sphingosine to S1P and which has been associated with mucosal inflammation in clinical samples and animal models (1, 27, 28). 24 h after injection of LPS, levels of SPHK1 were increased in inflamed ear pinnae (Figure 8A) suggesting inflammation drives the production of S1P. To investigate whether inflamed human tissues also have altered levels of S1P, we analyzed the levels of SPHK1 in biopsies taken from patients with the inflammatory autoimmune disease, RA, or from less inflammatory OA patients. Importantly, CD4 T cells are known to accumulate in RA synovial tissues and contribute to disease (29). As in the mouse samples, inflamed RA, but not OA, synovial tissues had raised levels of SPHK1 (Figure 8B).

## Discussion

T cell migration is a vital process for the immune system in homeostasis and following infection (2, 13). It enables T cells to navigate from lymphoid organs following activation to inflamed peripheral tissues. At inflamed sites, CD4 T cells can coordinate the clearance of pathogens but can also contribute to damaging chronic inflammation. Here, we have investigated factors that affect the persistence of activated CD4 T cells at inflamed sites. By taking a reductionist approach, we have focused on mediators involved in cell persistence rather than recruitment. An improved understanding of such factors has the potential to reveal targets to either enhance the persistence of activated CD4 T cells in peripheral tissues, for example in a vaccine setting, or reduce them in chronic inflammatory diseases such as RA.

We have found that inflammation increases the number of activated CD4 T cells in tissues. This increased persistence was not a consequence of increased proliferation and similar numbers of CD4 T cells were found in the draining LN and beyond. The similar ability to migrate to the draining LN from the resting or inflamed tissue suggests that inflammation increases the survival of CD4 T cells in the tissue itself. However, we were unable to directly demonstrate improved survival of the transferred OT-II T cells in inflamed ears, leaving the mechanism of the inflammation-induced increase in CD4 T cells currently unclear. Our attempts to identify dying CD4 T cells may have been hindered by the rapid clearance of apoptotic cells by tissue macrophages (30). What is clear is that S1P plays a critical role in promoting the persistence of activated CD4 T cells at sites of acute inflammation.

Sphingosine-1-phosphate is a pleiotropic molecule that has numerous effects on cells including proliferation, migration, cell adhesion and survival (31). Mendoza et al. (32) have recently reported a role for S1PR1 in enhancing the survival of naïve circulating CD4 T cells. Our results extend these data by demonstrating a key role for S1PR1 in regulating the number of activated CD4 T cells found in inflamed sites. Activated CD4 T cells treated with the S1P receptor antagonist, FTY720, move more slowly compared with control T cells. The low T cell velocity following either treatment with FTY720 (2.2 ± 2.2 μm/min) or transfer into non-inflamed ears (1.3 ± 1.6 μm/min) is similar to that of T cells at early stages of apoptosis *in vivo* (33). Together, these data indicate that inflammation-induced S1P may promote the survival of activated CD4 T cells.

By contrast, our finding that treatment of activated OT-II cells T cells with S1PR1 antagonists did not reduce the survival of these cells *in vitro*, suggest that S1P may not be acting as a survival signal in our *in vivo* model. Alternatively, S1P provides protection from an apoptosis signal present in the tissue itself. Future experiments will address the fate of the transferred T cells and determine whether S1P acts directly or indirectly to increase persistence of CD4 T cells at sites of acute inflammation by promoting cell survival and/or by other mechanisms.

Blocking S1PR1 either *via* FTY720 or the S1PR1 specific antagonist, W146, led to a reduction in the numbers of transferred OT-II T cells recovered from the ear without affecting the numbers of cells in the draining LN. These data conflict, in some instances, with previous publications that examined the migration of total spleen cells or activated CD4 T cells from resting or inflamed tissue sites (34, 35). A number of experimental details differ between our work and these previous studies. Most notably we have examined the injection site and the draining LN rather than only assessing cellular migration in the draining LN.

Ledgerwood et al. found the treatment of resting spleen cells with FTY720 or SEW2871 led to a reduction in the numbers of transferred cells recovered from the draining LN. The authors concluded that FTY720 acts as an agonist and that signals through S1PR1 inhibit cell migration (34). In our hands, FTY720 acts as an antagonist: the persistence of activated CD4 T cells in inflamed sites is inhibited following treatment with FTY720, PTX, and the S1PR1 specific antagonist, W146. Importantly, treatment of activated CD4 T cells with the S1PR1 specific agonist, SEW2871, before transfer, was sufficient to increase cell recovery from non-inflamed ears.

Recent studies suggest that downregulation of S1PR1 accompanies the differentiation of Trm cells (5, 6, 36). Our data could be interpreted to suggest that S1P signals may promote Trm generation *via* increasing survival of activated CD4 T cells in inflamed tissues. This is likely an over-simplification of the complex events culminating in Trm cell formation. Most significantly, our data are focused on very early time points while Trm differentiation is likely to occur in the weeks following a response. Our data do suggest, however, that disruption of S1P signaling early after T cell activation may counter rather than promote the generation of Trm cells.

Our studies have focused on examining the role of S1P in the context of Th1 cell persistence in acute and chronically inflamed tissues, but likely reflect a broader regulation of T cell activity at peripheral sites. The similar expression of S1PR1 on Th1 and Th2 cells suggests that Th2 cells are likely to be regulated in a similar fashion *in vivo* (37). Expression of S1PR1 is also required for the accumulation of regulatory T cells in tumors indicating that these cells may also be affected by S1P at inflamed tissues (38).

Currently, FTY720 is an approved treatment for multiple sclerosis and can reduce the onset of disease in animal models of inflammatory disease such as RA (39–43). In these approaches, systemic inhibition of S1P receptors is thought to reduce the entry of activated CD4 T cells into target tissues by preventing CD4 T cells from leaving lymphoid organs. Our finding that SPHK1 is increased in inflamed synovial tissue from RA patients suggests that S1P may also play a role in regulating the accumulation of activated CD4 T cells in human diseases. Indeed, there is a growing realization that S1P levels are increased in inflammation. S1P and S1P receptors are reported to be increased in synovial samples from RA compared with OA patients (44, 45). In addition, S1P is found at raised levels in the bronchoalveolar lavage of asthma patients and in intestinal tissue in preclinical animal models and clinical samples from individuals with inflammatory bowel disease (28, 46). Chronic inflammation is a multifactorial process involving a plethora of different cell types and inflammatory molecules (47). Our current *in vivo* data are limited to an acute model of inflammation in mice. Our future studies will investigate whether S1P influences the switch from acute to chronic disease and the maintenance of inflammation by promoting activated CD4 T cell persistence in these settings.

In summary, we have shown that acute inflammation promotes the persistence of CD4 T cells at tissue sites and that signals through S1PR1 are both necessary and sufficient for this maintenance. An increased understanding of how S1P mediates this effect could reveal opportunities to manipulate the levels of S1P in peripheral tissues to promote or reduce survival and persistence of activated CD4 T cells.

## Ethics Statement

Animal experiments were covered by a Project License granted by the UK Home Office under the Animals (Scientific Procedures) Act of 1986 and approved by the University of Glasgow Ethical Review Committee. Synovial tissue specimens were obtained from RA and osteoarthritis (OA) patients during arthroscopic biopsy or total joint replacement surgeries at Glasgow Royal Infirmary (Glasgow, UK). All RA and OA patients fulfilled the diagnostic criteria for RA and OA classification, respectively, and written consent form was obtained from all subjects. All procedures received Ethics Approval (West of Scotland Research Ethical Committee Approval: 11/S0704/7).

## Author Contributions

SJ designed the research, acquired and analyzed the data, and drafted the article. RB acquired and analyzed data and revised the article. AE acquired and analyzed data. MK-S designed experiments and interpreted data. IM and PG designed experiments and edited the article. MM conceived and designed the research, analyzed data, and drafted the article.

## Conflict of Interest Statement

The authors declare that the research was conducted in the absence of any commercial or financial relationships that could be construed as a potential conflict of interest.
