# Supplementary material for: Sphingosine-1-Phosphate Promotes the Persistence of Activated CD4 T Cells in Inflamed Sites
Source: Front Immunol. 2017 Nov 24;8:1627. doi: 10.3389/fimmu.2017.01627 (PMC5705559; doi:10.3389/fimmu.2017.01627)
Supplement: Supplementary file 1 [file Data_Sheet_1.PDF]

Supplementary Figure 1

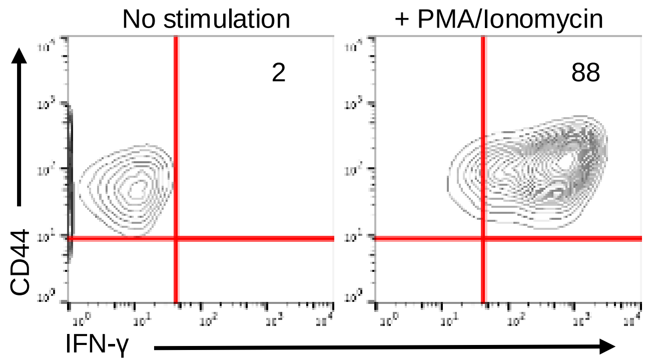

Supplementary Figure 1: Th1 cell differentiation culture conditions induced a high level of cell differentiation

Naïve CD4 OT-II T cell isolated by negative selection were activated with ovalbumin peptide 323-339 *in vitro* in the presence of mitomycin C treated spleen cells, IL-12 and anti-IL 4 for three days. To assess the extent of Th1 cell differentiation, these cells were activated with PMA and ionomycin for 4 hours in the presence of golgi plug and then analysed by intracellular flow cytometry. Cells are gated on CD4+ T cells and the number shows the percentages of these cells that were CD44+ IFN-γ+. Data are representative of more than 10 experiments.
