# Supplementary material for: Sphingosine-1-Phosphate Promotes the Persistence of Activated CD4 T Cells in Inflamed Sites
Source: Front Immunol. 2017 Nov 24;8:1627. doi: 10.3389/fimmu.2017.01627 (PMC5705559; doi:10.3389/fimmu.2017.01627)
Supplement: Supplementary file 2 [file Data_Sheet_2.PDF]

Supplementary Figure 2

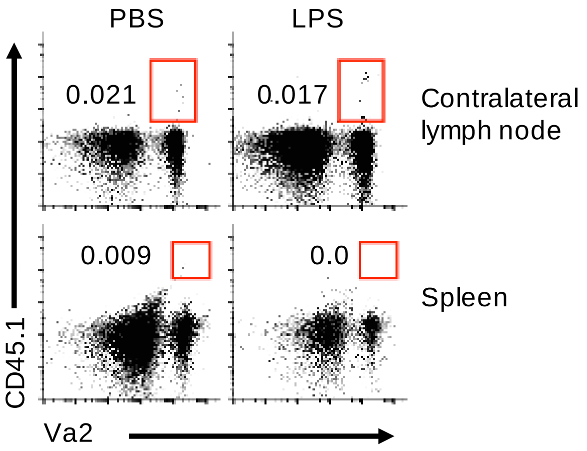

Supplementary Figure 2: Few transferred activated OT-II T cells were found in lymphoid organs downstream of the cervical lymph node

Activated OT-II T cells were injected into mouse ears that had been injected with PBS or LPS 24 hours previously. After a further 24 hours, the percentages of CD45.1+ OT-II T cells present in contralateral superficial cervical lymph nodes and spleens were analysed by flow cytometry. Data are representative FACS plots from 2 experiments with ≥3 animals per group. Numbers show the percentages of OT-II T cells out of total live CD45+ cells.
