# Supplementary material for: Sphingosine-1-Phosphate Promotes the Persistence of Activated CD4 T Cells in Inflamed Sites
Source: Front Immunol. 2017 Nov 24;8:1627. doi: 10.3389/fimmu.2017.01627 (PMC5705559; doi:10.3389/fimmu.2017.01627)
Supplement: Supplementary file 3 [file Data_Sheet_3.PDF]

Video 1: T cells movement *in vivo* is restricted in PBS-injected ears

Th1 differentiated DSRed OT-II T cells were injected into the ear of a CD11c-YFP mouse that had been injected with PBS 24 hours previously. The injected ear was imaged 6 hours later. The video is representative of three individual animals. Transferred OT-II T cells are in red, CD11c+ cells in green and the collagen network is visible via second harmonics in blue. Scale bar represents 50μm.

Video 2: T cells movement *in vivo* is non-direction in LPS-inflamed ears

Th1 differentiated DSRed OT-II T cells were injected into the ear of a CD11c-YFP mouse that had been injected with LPS 24 hours previously. The injected ear was imaged 6 hours later. The video is representative of three individual animals. Transferred OT-II T cells are in red, CD11c+ cells in green and collagen network visible via second harmonics in blue. Scale bar represents 50μm.

Videos 3 and 4: T cells movement *in vivo* is reduced following pre-treatment with FTY720

Th1 differentiated DSRed OT-II T cells were treated with vehicle (Video 3) or FTY720 (Video 4) before injection into the ears of mice that had been injected with LPS 24 hours previously. The injected ear was imaged 6 hours later. The video is representative of three individual animals. Transferred OT-II T cells are in red and collagen network visible via second harmonics in blue. Scale bars represent 50μm.
